# Medical student resilience strategies: A content analysis of medical students’ portfolios

**DOI:** 10.1007/s40037-016-0313-1

**Published:** 2016-12-12

**Authors:** Richard A. Prayson, S. Beth Bierer, Elaine F. Dannefer

**Affiliations:** 1 Cleveland Clinic Department of Anatomic Pathology, Cleveland, OH USA; 2 Cleveland Clinic Lerner College of Medicine of Case Western Reserve University, Cleveland, OH USA

**Keywords:** Resilience strategies, Portfolio assessment, Undergraduate medical education, Personal development

## Abstract

**Introduction:**

Stress and burnout among medical students is a well-recognized concern. A student’s ability to employ resilience strategies to self-regulate behaviour is critical to the student’s future career as a physician.

**Methods:**

We retrospectively reviewed a sampling of year 1, 2 and 5 portfolio essays focused on the Personal Development competency and performance milestones, written by 49 students from three different classes in a 5-year programme devoted to training physician investigators. Two medical educators used a framework established by Jensen and colleagues (2008) to identify the nature and prevalence of various resilience strategies (valuing the physician role, self-awareness, personal arena, professional arena, professional support and personal support) medical students reported in portfolio essays.

**Results:**

All students documented at least one strategy in their essays each year. In all years, the most commonly documented strategies were in the personal arena (95.7% of year 1, 98% of year 2 and 87.8% of year 5 portfolios). The least frequently documented strategy in all years was professional support (42.8% of year 1, 38.8% of year 2, and 28.6% of year 5 portfolios). Year 5 portfolios discussed personal support strategies (79.6%) more frequently than year 1 (53.1%) and year 2 (59.2%) portfolios.

**Discussion:**

The results suggest that medical students can identify stressors and articulate resilience strategies that can be employed to potentially address them.

## What this paper adds

- A medical student’s ability to utilize resilience strategies to self-regulate behavior is important to the student’s future career as a physician. Educational strategies to facilitate this are important. This study examines the use of a Personal Development competency in a portfolio assessment system to engender reflection on resilience strategies. The study shows that students, with mentorship, are able to identify stressors and articulate resilience strategies to address those stressors.

## Introduction

Medical school training is well known to be stressful and associated with a higher than normal risk for burnout [1]. Pressures to perform, heavy workloads, struggles to maintain life balance, limited control, and the unique transformative nature of experiences (i. e. dissecting cadavers, physical exams on patients, dealing with life and death issues with patients, etc.) are but a few of the contributing factors. In addition to dealing with stress directly generated by school experiences, students often, like the rest of us, have to cope with personal life stresses. Hojat and colleagues (1999), in studying 1157 first and second year medical students at one school, noted that 42% of students experienced financial problems, 36% experienced a change in the health of a relative, 26% experienced a personal illness or injury, and 15% experienced a death in the family [2]. Beside personal life issues, studies have examined a variety of other aspects and their relation to student stress, including issues of gender, marriage and children, ethnicity, and personality [1]. It is well known that these student stresses predispose to higher risks of depression, anxiety, substance abuse, struggles with interpersonal relationships, suicide, and burnout (emotional exhaustion, depersonalization and a sense of low personal accomplishment) [3, 4]. Student stressors have also been shown to impact professional effectiveness by contributing to problems with attention, concentration, cynicism, academic dishonesty, poor decision-making and communication skills [3, 4]. The associated risks and impacts of stress on professional life are not just confined to medical school training; they continue during residency and in the subsequent practice of medicine [5, 6]. A recent longitudinal study of 6880 physicians found that 54.4% of physicians reported at least one symptom of burnout in 2014 (up from 45.5% in 2011) and that satisfaction with work-life balance declined from 2011 (48.5%) to 2014 (40.9%) [7].

The literature documenting stressors associated with medical student education is sizeable. Shapiro et al. (2000) found over 600 articles published from 1966–1999 on the subject [3]. However, the literature documenting interventions to prevent distress in medical students is much more limited. They found only 24 studies of the over 600 papers reviewed that reported intervention programmes; only six of these were judged to have employed a rigorous scientific methodology. Schools that did have stress-management programmes demonstrated decreased depression and anxiety among their students, improved knowledge of the effects of stress, a greater utilization of coping strategies, and a better ability to resolve role conflicts. There is some suggestion, in the physician-focused literature, that coaching and supportive relations may contribute to more successful adaptation [8, 9]. A need exists to explore the resilience strategies medical students employ to address this challenge.

In 2004, the Cleveland Clinic Lerner College of Medicine (CCLCM) programme of Case Western Reserve University five-year medical school programme (32 students per class) matriculated its first class with the goal of training physician scientists. Instead of utilizing a traditional, grade-based assessment system for student performance, a portfolio assessment process was adopted to document student performance in nine competencies [10]. For each competency, students are asked to reflect on their performance and formative feedback and self-assess how they are doing with regard to performance standards or milestones. The process is carried out in dialogue with a physician advisor, who serves as a mentor and reality check for the student. Students are encouraged to construct a learning plan [11] in order to address performance deficits and monitor progress. Over the course of the five years, students submit a series of formative portfolios and/or learning plans to their physician advisor for review and discussion. Additionally, at the end of years 1, 2, and 4, students submit summative portfolios to a promotion committee for assessment of progress with regard to meeting competency milestones.

Of the nine CCLCM defined competencies, most of which map to the Accreditation Council for Graduate Medical Education (ACGME) residency competencies, the Personal Development competency is the most unique. This competency is critical in being able to optimally master the other eight competencies; if our future physicians are unable to take care of themselves in the arenas of personal health, stress and time management, and life-balance, how can they do their best job in taking care of patients? Prompts provided to students to facilitate reflection on the milestones in this competency ask students to (1) critically reflect on personal values and priorities with the goal of developing strategies to promote personal growth and (2) identify challenges between personal and professional responsibilities and develop strategies to deal with them. Portfolios provide opportunities for students to self-reflect on their medical school and personal life experiences as they relate to this competency. Our experiences, as a physician advisor since the inception of the programme (RAP) and Director of Student Assessment (EFD), have provided multiple opportunities to review students’ portfolios and note that students discussed resilience strategies in the venue of the Personal Development competency essays.

CCLCM’s assessment system’s emphasis on Personal Development provides a unique opportunity to identify the resilience strategies medical students adopt to alleviate stressors and develop work-life balance. This study uses a retrospective, content analysis of students’ portfolios to explore resilience strategies students employ at different time points to self-regulate their behaviours for the Personal Development competency.

## Methods and materials

Institutional Review Board (IRB) approval was obtained prior to commencement of the study.

Forty-nine students were randomly selected in an anonymous fashion from three classes (graduating classes of 2011, 2012 and 2013) with 17 students from the class of 2011, 16 students from the class of 2012 and 16 students from the class of 2011 forming the study group; this corresponds to approximately half of students in each of these classes which was felt to provide a fair representation of students’ perspectives. The Personal Development competency essays from the year 1, year 2 and year 5 summative portfolios were extracted and anonymized by a research coordinator for each of the study subjects. A directed content analysis [12] of the portfolios was performed utilizing a previously defined framework for examining resilience strategies in physicians [9]. Each essay was independently reviewed by two experienced medical educators (RAP, EFD), looking for documentation of resilience strategies. The strategies were coded into one of six general categories (valuing the physician role, self-awareness, personal arena, professional arena, professional support and personal support) based on previous work describing attributes and approaches in building resilience in physicians by Jensen et al. (2008) [9]. The types of strategies included with each of the six categories are summarized in Table 1. In instances where there was a discrepancy in coding, the issue was discussed until an agreement was reached; discrepancies in coding occurred in less than 2% of instances. The Appendix to this paper provides a sampling of quotes taken from various portfolio essays for each of the six coding categories.

In writing their portfolio essays, students are encouraged to cite specific evidence from feedback they have received to substantiate their discussion. Feedback evidence most commonly originated from written assessments provided by faculty and peers [13]. Students are encouraged to also be creative and are permitted to employ other sources of evidence such as emails, reflective pieces they may have written for course work, copies of schedules, etc. The number of pieces of cited evidence for each essay was also documented.

## Results

Table 1 summarizes the results of the resilience strategy coding for the 49 reviewed portfolios organized by the student’s year in training during which the portfolio essay was written. All strategies were documented in a subset of portfolio essays in each year. In all years, the most frequently documented strategies were in the personal arena (ranging from 87.8% of year 5 portfolios to 98.0% of year 2 portfolios). In their portfolios, pertinent to this category, students described their extracurricular activities. This included school-related activities, as well as endeavours outside of school, including recreational activities and a wide assortment of personal hobbies. Students also sometimes discussed vacations as part of taking time for themselves and as an opportunity to spend time with family or friends. Less frequently, students discussed their religious beliefs and/or practices as being important to them. Students frequently mentioned exercise, nutrition and sleep-related issues.

**Table 1:** Summary of resilience strategies documented on portfolios (*n* = 49 students)

Strategy	Year 1	Year 2	Year 5
Valuing physician role	*n* = 25 (51.0%)	26 (53.1%)	31 (63.3%)
Self-awareness	27 (55.1%)	26 (53.1%)	21 (42.8%)
Personal arena	47 (95.9%)	48 (98.0%)	43 (87.8%)
Professional arena	43 (87.8%)	44 (89.8%)	42 (88.7%)
Professional support	21 (42.8%)	19 (38.8%)	14 (28.6%)
Personal support	26 (53.1%)	29 (59.2%)	39 (79.3%)

The least cited strategies were those related to professional support in the school environment (ranging from 28.6% in year 5 essays to 42.8% in the year 1 essays). In this arena, students valued the support they received from their peers. They discussed the importance of particular mentors who helped them either in a professional or personal capacity. Rarely did students discuss seeking help from health care professionals in the management of personal, health-related issues.

The most notable difference between 1st and 5th year portfolios was with documentation of personal support strategies, which were much more commonly discussed in year 5 portfolios (79.6%) versus year 1 portfolios (53.1%). Students commonly discussed the role partners, significant others, family and friends play as support systems. Students also occasionally recognized other individuals such as personal physicians or religious figures outside the immediate school environment as sources of support.

The majority of students in all years (>85%) discussed the importance of activities within the professional arena as useful strategies. Many of these centred around activities or curriculum in the school around a variety of aspects of medical school life and included issues such as time management, pursuing a master’s degree, shadowing physicians in order to help with career decision making, skills’ building in the communication arena, career development, assuming leadership roles in various student organizations as well as in activities outside the school venue, reflecting on how to set limits, and strategies focused on how to deal with board exam preparation.

Roughly half of students across all years discussed valuing the physician role and self-awareness issues in their portfolios. Discussions around valuing the physician role focused on subjects of maintaining interest in being a medical student committed to a career as a physician, the importance of contribution to the field of medicine and to patient care, struggling in learning to accept the professional demands of their career choices, the appreciation of the privilege and implications of that responsibility in their current roles and future roles as physicians, learning how to interface with the lives of others (especially patients), maintaining enthusiasm about their patient care role, teaching patients, issues of work ethic and professionalism and extending outside the hospital setting to serving others in the capacity of volunteer work or community service projects.

Discussions around the subject of self-awareness focused on topics of accepting personal limits, stress management, dealing with health issues, finances, self-regulation and self-reflection, and issues related to reflective/portfolio writing itself.

All students formally cited at least one piece of evidence to support their discussions in each essay. The mean number of cited pieces of evidence decreased from 1st to 5th year portfolios: year 1 portfolios – mean of 8 pieces of evidence cited (range 1–31); year 2 portfolios – mean 6 pieces of evidence cited (range 1–30); and year 5 portfolios – mean 5 pieces of evidence cited (range 1–13).

## Discussion

The purpose of the current study is to explore resilience strategies students employ in self-regulating their behaviours in the Personal Development arena, as documented in portfolios for different levels of learners. The portfolio provides a tool by which students can engage in reflective practice and provides a potential vehicle for open discussion and possible intervention for students with respect to resilience strategies to address various stressors. Engaging in reflective practice creates an opportunity for the learner to pause and consider varying perspectives, while maintaining an open mind [14]. Reflective practice requires being engaged in actively processing, analyzing and synthesizing experiences and feedback. It challenges one to examine goals, practices and beliefs. Its purpose is to gain insights which culminate in action. To this end and in reflecting on Personal Development, students are encouraged to consider the following: 1) Why do you do what you do? 2) What are the implications of what you do (both immediate and long-term)? 3) Why is it important to change? 4) What is the best way to effect change? and 5) How do you know whether your approach to change is working or not?

Given that it is important to have oversight in high stakes assessments, the student’s physician advisor is an integral part of the CCLCM learning environment and plays a key role in facilitating and monitoring this process, reality checking a student’s perceptions, providing a safe person with whom issues and concerns can be vetted and ensuring an ongoing dialogue around the competencies. Inclusion of Personal Development as a competency on par with eight other competencies (medical knowledge, communication, research, professionalism, interprofessional collaboration, clinical reasoning and skills, health care systems, and reflective practice) sends the message that consideration of this is important; after all, we assess what we value. Milestones provide guidelines which allow students to monitor development in the competency over time.

The current study demonstrated that many students are indeed cognizant of some of the stressors associated with their role and can identify resilience strategies to employ in dealing with some of these stressors. This study also suggests that many students are able to employ external feedback (as documented by cited evidence) to help identify potential issues in this arena, although not all to the same extent.

Not surprisingly, resilience strategies documented in year 1 and 2 are similar. The classroom environment in the first two years of school, which serves as fodder for the first two summative portfolios, creates a relatively stable and predictable learning environment upon which to reflect. The year 5 summative portfolio follows both the clinical rotations and a year of research. The impact that these experiences may have on students’ perceptions and reflections may explain some for the shift in content focus observed in the portfolios. The most notable change seen was increased documentation of personal support strategies. It is conjectural to say what accounts for these differences and there are a variety of factors that might account for this, including experiences related to the stresses of clinical clerkships and rotations, changes in class dynamic and learning environment as the student cohort moves from the classroom and are scattered to hospital or research laboratory learning environments, effects of research year experiences, and personal life circumstances (e. g. increasing numbers of students in relationships and starting families). This shift in learning environment may also explain, in part, the downward trend in documentation of professional support. Class attendance in the first two years of the programme is mandatory, which results in all 32 students spending all their class time together. During clerkships and especially during the research year, they do not have nearly as much contact on a daily basis with their peers and opportunities for peer support are less common. This shift may also account for the slightly decreased documentation of personal arena issues; there is less time available during clinical rotations for hobbies and extracurricular and recreational activities. More interaction with patients and physicians during clerkships likely engenders students to give more consideration of their roles as caregivers and physicians-to-be.

There are a number of limitations to the current study. The structure of the programme and its assessment system are somewhat unique among medical school programmes; the impact of the learning environment created, as a result, could impact what and how students document in their portfolios. The portfolio essay, due to word restrictions, may not fully capture all resilience strategies students employ. Documentation does not necessarily guarantee implementation. Although mentors play an important role in helping students learn how to process feedback and reflect, biases of perspective may be introduced by the advisors which may impact what students feel they should or do not need to include in their portfolios.

The goal of using the portfolio assessment approach is to promote self-regulation, a skill which entails monitoring, self-assessment, and modulation of performance via goal setting and implementation of strategies to improve [15]. As future physicians, self-regulation of learning and behaviour, how the learner understands social values and extrinsic contingencies and transforms them into a personal value system and self-motivation [16] are critical skills in meeting performance standards for the profession. The portfolio provides a potential instrument for encouraging students to reflect on stressors they are currently experiencing and may experience in the future as practising physicians and identify potential strategies to mitigate some of these stressors. The ability to internalize this practice will hopefully arm students as future physicians in dealing with issues of stress and burnout that unfortunately plague many practising physicians in the current medical climate. It would be interesting to know how strategies shift, change or evolve/devolve at transition points over time, if at all, e. g. as students transition from medical school to residency and from residency to staff.

## Appendix

### Sample of quotes from portfolios documenting resilience strategies

#### Valuing physician role

“… a large part of the challenge I experienced when I first hit the clinics was the dichotomy between my ‘doctor-self’ and my actual self. I strived to bring the two closer together as best as possible by taking a ‘humanistic’ approach in trying to not just express empathy, but truly experience it.”

“Based upon observations made while growing up, I developed an appreciation for and familiarity with the challenges facing the lower socioeconomic classes. Because of this, I always ensure to make the most of the opportunities I seize. As a result, I strongly feel I should utilize my resources to help benefit others, specifically those less fortunate than myself. By assisting these individuals, I feel I can potentially increase their abilities to one day take advantage of opportunities that may arise. Being a medical student, however, my resources to draw upon are currently quite limited. This restriction, only applies to my financial assets though, and not my continually growing skill set and knowledge base.”

#### Self-awareness

“And although I have seen many of my preceptors get involved on so many levels, I seemed to notice that those that were the happiest (and perhaps most successful) were the ones that also maintained their personal interests and a healthy family life. I have placed a greater priority on making sure to keep up with my hobbies …”

“Perhaps the most important conclusion that I have come to over the year is the need for continual improvement as a member of the medical field. The field of medicine rapidly evolves and new and exciting discoveries are made daily. It is important to stay current on these discoveries on a continual basis through the medical literature to provide the best possible care to patients in the future. Constructive criticism is also important to continual improvement. It is difficult, if not impossible, to demonstrate growth and development without objective comments from others. These serve as invaluable tools to development and growth in all competencies. Assessments from others also provide a framework to quantitatively track progress and improvements. I have been open to constructive criticism throughout the year and strongly feel that I have recognized and improved upon various areas for improvement as a result. Lifelong learning and continual improvement will only make me a better physician in years to come.”

#### Personal arena

“I am trying to focus more on my responsibilities to myself, such as running in the half-marathon and finding and moving into my own apartment … This can be challenging, especially in a curriculum with so few external milestones by which one can meter ones’ own performance – internally you can always tell yourself that you could have done more, if only you had studied or read or worked on learning objectives and not done your full run, or gotten a little less sleep, or not kept in touch with your friends from before. These things are important to do anyways, however, as they are part of what makes us complete human beings.”

“At the end of third year, I was ready for a change. I was happy to have completed all of my required clerkships, but my efforts to be a good student while maintaining my personal life had left me exhausted. Through a lens of fatigue, I was disturbed to feel my empathy decreasing with every overnight call on my last rotation. I still had a deep-seated desire to be good at my job, but my love for the day-to-day grind had dulled after a long year.”

#### Professional arena

“… still struggling to find a balance between degree of structure that will facilitate my accomplishing all of my tasks and a degree of flexibility that best suits my personality and learning style … I tried a new strategy of implementing a small number of structured activities that I could flexibly arrange other activities around. I found that introducing this small number of realistic changes was very effective at imitating behavioral modifications and promoting long-term adherence. While these targeted interventions have proved beneficial, I have also come to realize that there is a natural maturation occurring as I continually adjust to life as a medical student. Inherent to this maturation is a more instinctive ability to identify and manage time conflicts without formal recognition and intervention.”

“I have developed strategies to effectively deal with a workload that would have seemed overwhelming had I not grown accustomed to it. I have learned to schedule ahead, set aside time for studying, and give myself time limits for working. I have also grown comfortable with designating time to spend with others as well as relax on my own – essentially I have learned to be at ease with not working despite having a lengthy queue of tasks awaiting completion. Additionally, I gained experience identifying and estimating what is required of me and honestly informing those in my life of my availability.”

#### Professional support

“Recently, however, I have realized that my education and personal growth are more important than my pride or my fear of stigma. I have begun to take my own mental health seriously, starting medication and being careful to get exercise, write in a journal, and seek a supportive social network.”

“I recognize that along my training there will undoubtedly be times where my family will need me. Though there is no way to prepare for these circumstances, as long as I continue to keep my peers and faculty informed and uphold my responsibilities as best as possible, these times will pass. Just as my family and community rely on me, I know that in times of need I can always rely on them for support.”

#### Personal support

“This past year has been difficult for my family, because my [relative], who has been ill … passed away … Having observed the rapid decline he went through, I became keenly aware of what it means to be a family of a dying patient. One of the challenges I faced was separating my emotional response from patient care. Within a week of … death, I was working as an acting intern in family medicine inpatient service, taking care of a young woman who was found to have recurrence of breast cancer with extensive metastases. I tried to advocate for the patient to be able to go home in between her whole brain radiation and the initiation of her chemotherapy. However, the rest of the team felt that it was safer for her to stay in the hospital, as she would receive appropriate medical care. This was a difficult decision for me to advocate for, because I knew how important it can be for families to be able to spend uninterrupted time at their end of their lives. However, I had to recognize my personal biases, and separate my personal opinions and emotions from professional responsibility. Through the experience, I have become cognizant of how my personal experiences can influence clinical decision making, and learned to keep an objective focus on professional responsibilities first.”

“I have developed some strategies to promote personal growth both within and outside my medical career. Knowing my priorities is helpful in that I ‘keep my eye on the ball’and stay on track with that is important to me. My [spouse] and I have regularly gone through an exercise of writing out our personal goals together, and then organizing them into a chart that we post around the house to remind us of our priorities, values and goals. We have done this with many different areas of our life such as family, marriage, volunteerism, spiritual, financial and career. This has been incredibly helpful in moving us toward reaching the goals that we feel are necessary to living a meaningful and satisfying life.”
